# A New VISTA on combination therapy for negative checkpoint regulator blockade

**DOI:** 10.1186/s40425-016-0190-5

**Published:** 2016-12-20

**Authors:** Jie Deng, Isabelle Le Mercier, Anna Kuta, Randolph J. Noelle

**Affiliations:** 1 Department of Microbiology and Immunology, Geisel School of Medicine at Dartmouth, Norris Cotton Cancer Center, One Medical Center Drive, Room 730, Lebanon, NH 03756 USA; 2 ImmuNext, Inc., One Medical Center Drive, Lebanon, NH 03756 USA

**Keywords:** Negative checkpoint regulators, Combination immunotherapy, VISTA

## Abstract

Negative checkpoint regulators function to restrain T cell responses to maintain tolerance and limit immunopathology. However, in the setting of malignancy, these pathways work in concert to promote immune-mediate escape leading to the development of a clinically overt cancer. In the recent years, clinical trials demonstrating the efficacy of blocking antibodies against these molecules have invigorated the field of immunotherapy. In this review, we discuss the current understanding on established NCR blockade and how strategic combination therapy with anti-VISTA antibody can be used to target multiple non-redundant NCR pathways.

## Background

Negative checkpoint regulator (NCR) blockade has begun to establish itself as a cornerstone to multi-modality cancer treatment. Pioneered by seminal findings in multiple mouse models and human cancers, monoclonal antibody blockade of Cytotoxic T Lymphocyte Antigen 4 (CTLA-4) has paved the way for the field of NCRs leading to discovery of new molecules as well as uncovering novel approaches for combination therapy targeting multiple non-redundant pathways. In this review, we describe the prevailing wisdom of mechanisms of action for established NCR blockade. Further, we discuss the role of V-domain Ig Suppressor of T Cell Activation (VISTA), a novel target in immuno-oncology. Alongside the phase 1 clinical trial testing anti-VISTA (NCT02671955), it becomes increasingly important to have a comprehensive understanding of how targeting VISTA advantageously shapes the tumor microenvironment.

### Blockade of CTLA-4 paving the way for checkpoint blockade

CTLA-4 is exclusively expressed on T cells, although not on naïve or memory T cells. CTLA-4 has two binding partners, B7 molecules CD80 and CD86, both of which are expressed on antigen presenting cells (APCs). CTLA-4 exerts its immunosuppression of T cell responses by multiple mechanisms. First, CTLA-4 outcompetes the co-stimulatory molecule CD28 for their shared binding partners CD80 and CD86 as a result of increased affinity, increased avidity, and more stable interactions within the immunological synapse [1–6]. In addition, upon disrupting co-simulation by CD28 and ligation with cognate B7 molecules, CTLA-4 recruits the inhibitory tyrosine phosphatase SHP-2 to the T cell receptor, thus attenuating the signal [1, 2, 7], destabilizing the immunological synapse [1, 8], arresting cell cycling [9], and reducing the production of IL-2 [1, 2, 7, 9]. CTLA-4 can also signal bi-directionally to induce the upregulation of indoleamine 2,3-dioxygenase (IDO) by APCs [10, 11]. In vivo, it is hypothesized that the predominant mechanism through which CTLA-4 downregulates T cell response is via transcytosis of CD80 and CD86 from the membranes of APCs, thus making these molecules unavailable for co-stimulation by CD28 [1, 8, 12]. Furthermore, natural FoxP3^+^ regulatory T cells (nTreg) constitutively express high levels of CTLA-4 on their surface and both CTLA-4 on nTregs as well as conventional T cells contribute to T cell immunosuppression [13].

The major role CTLA-4 plays in restraining T cell responses to maintain tolerance is evident in mice that have a complete deficiency of CTLA-4 [1, 9, 14, 15]. Within a few weeks, CTLA-4^-/-^ mice die from massive, spontaneous, lethal autoimmunity due to unchecked lymphoproliferation [14, 15]. Activated T cells aggressively infiltrated spleen and lymph nodes as well as the liver, heart, lung and pancreas with high immunoglobulin detected in the blood [14, 15]. This severe and widespread autoimmune phenotype suggests that CTLA-4 primarily functions to control T cell responses for maintaining immune homeostasis. Importantly, the absence of CTLA-4 specifically on Tregs is sufficient for the development of autoimmunity, further reiterating the major role of CTLA-4 in Treg mediated suppression [16].

These observations that CTLA-4 functions to restrain T cell response led to the hypothesis that blockade of CTLA-4 may lead to persistent T cell activity within tumors. Success in multiple pre-clinical models [17, 18], led to the development of Ipilimumab (Bristol-Myers Squibb), a fully human IgG1 monoclonal antibody and first in class of NCR blockade. After two transformative phase III trials in melanoma [19, 20], Ipilimumab received FDA approval in 2011 for the treatment of advanced melanoma. Further, durable immunity was established evident by responses lasting up to 10 years in some patients [21]. The mechanisms underpinning the in vivo efficacy of CTLA-4 blockade remains controversial. Targeting both effector T cells and Tregs appear critical for maximal anti-tumor activity [22]. However, anti-CTLA-4 activity has also been linked to Fc mediated Treg depletion at the tumor site by tumor infiltrating macrophages [23, 24].

### Blockade of PD-1/PD-L1 axis expanding boundaries

Similar to CTLA-4, Programmed cell death 1 (PD-1) is not expressed on naïve or memory T cells but is expressed on activated and exhausted T cells [25, 26]. PD-1 has two binding partners, Programmed cell death ligand 1 (PD-L1) and Programmed cell death ligand 2 (PD-L2). Though the role of PD-L2 as a target in immuno-oncology has yet to be clarified [27, 28], ample pre-clinical and clinical data demonstrate the relevance of PD-L1. PD-L1 is expressed on dendritic cells, macrophages, myeloid-derived suppressor cells (MDSCs), fibroblasts, and T cells [28–30]. PD-L1 has also been detected in multiple human cancer cells including breast cancer, colon cancer, glioblastoma, non-small cell lung cancer, melanoma, and ovarian cancers, among others [29]. In addition, some tumors such as melanoma express PD-1 which can directly promote tumorigenesis in a cell-intrinsic fashion by modulating mTOR pathways. Therefore, anti-PD-1 antibodies can also directly inhibit tumor growth [31].

Unlike CTLA-4, PD-1/PD-L1 engagement does not interfere with co-stimulation but negatively regulates anti-tumor immunity through apoptosis and inhibition of T cell signaling. Tumor cell expression of PD-L1 ligation triggers apoptosis of activated T cells leading to the loss of T cells in vivo and subsequent outgrowth of tumor [32–34]. Ligation of PD-L1 and PD-1 negatively regulates T cell activity by multiple ways. The cytoplasmic domain of PD-1 includes a conventional immunoreceptor tyrosine-based inhibition motif (ITIM) and immunoreceptor tyrosine-based switch motif (ITSM) which bind inhibitory tyrosine phosphatase SHP-2 [33], disrupts TCR-induced stop signal [35], and promotes T cell motility to limit the time of cognate interactions [36].

In contrast to CTLA-4 ^-/-^ mice, PD-L1^-/-^ [32] and PD-1 ^-/-^ [37, 38] mice do not develop massive, lethal lymphoproliferative disease. Instead, there is only organ-specific inflammation further supporting the fact that CTLA-4 and PD-1 function at different stages of immune activation.

The prior success of anti-CTLA-4 led to accelerated development of antibodies targeting PD-1 [39, 40] and PD-L1 [41]. Studies testing these new drugs uncovered several distinctions from anti-CTLA-4 therapy. In addition to enhanced efficacy in melanoma [39–41], disrupting the PD-1 pathway demonstrated efficacy in non small cell lung cancer [39, 41, 42], which was previously thought to be refractory to immunotherapy. Second, treatment with drugs blocking PD-1/PD-L1 caused fewer immune related adverse events than anti-CTLA-4, which is consistent with the less severe inflammatory phenotype of PD-1 and PD-L1 deficient mice compared to that of CTLA-4 deficient mice. Several drugs targeting either PD-1 (Nivolumab, Bristol-Myers Squibb; Pembrolizumab, Merck) or PD-L1 (Atezolizumab, Roche) have received accelerated FDA approval for various applications and more are in development.

### Maximizing response with combination therapy

Though a subset of patients responds to NCR blockade as monotherapy, the majority of patients do not respond to the release of a single axis. Thus, combination therapy targeting multiple pathways may provide additional support to the development of anti-tumor host immunity. Indeed, combination of drugs targeting CTLA-4 or the PD-1/PD-L1 pathway led to synergistic efficacy versus monotherapy in both murine models and patients [43–45] leading to FDA approval of combination Ipilimumab and Nivolumab for advanced melanoma.

Synergist clinical activity with combination therapy indicate non-redundant mechanisms of action for anti-CTLA-4 and anti-PD-1, which may be attributed to differential regulatory mechanisms dictating target expression. Expression of CTLA-4 is induced by TCR signaling [9] and surface expression is rapidly upregulated by mobilizing large intracellular pools of CTLA-4. Combined with the observation that its ligands CD80 and CD86 are expressed on APCs, it is believed that the CTLA-4 axis works to restrain T cells within secondary lymphoid organs where T cell activation occurs [1, 19, 20]. In contrast to CTLA-4, it is viewed that PD-1/PD-L1 pathway acts to restrain T cell responses in the peripheral tissues, such as at the tumor bed where ligand and receptor are both in abundance [1, 34, 46, 47]. PD-L1 can be upregulated by tumor cell oncogenes [48–53] or by other mediators highly expressed within the tumor microenvironment (TME) such as tumor hypoxia [30], TLR-mediated signaling pathways [54], and IFNγ [55]. As a result, future studies identifying regulatory mechanisms governing NCR expression may help guide strategic combination therapy and also aid in selecting patients to optimize treatment protocol.

Another approach to improving the response to NCR blockade, is reversing mechanisms which limit its efficacy. There are multiple mediators which subvert the full potential of immunotherapy such as tumor-infiltrating MDSCs. MDSCs are a group of heterogeneous, immature myeloid cells that are aggressively expanded and pathologically activated by tumor-derived factors [56]. MDSCs exert suppression over T cells through multiple mechanisms including the production of reactive oxygen species, nitric oxide, and arginase [57]; ultimately leading to T cell suppression [58] and increased tumor burden [57].

Importantly, the accumulation of MDSCs within the tumor bed has emerged as an important mechanism of resistance to immunotherapy such as NCR blockade. In combination anti-PD-1 and anti-CTLA-4 in B16 melanoma, reduced treatment efficacy was observed when MDSCs were recruited via IDO overexpression (B16-IDO) by the tumor [59, 60] and blocking MDSCs recruitment via CSF-1R blockade improved efficacy in B16-IDO but not in B16. In CT26 colon carcinoma model, characterized by its aggressive MDSC compartment [61], MDSC depletion enhanced the efficacy of the combination therapy of anti-CTLA-4 and anti-PD-1. While late intervention of anti-PD-1 and anti-CTLA-4 on large, established CT26 colon cancer and 4 T1 breast cancer did not have any therapeutic efficacy, supplementing the combination therapy with 5-azacytidine and etinostat to inhibit MDSCs, sensitized tumors to NCR blockade leading to tumor reduction [62]. Together, these studies clearly demonstrate that targeting MDSCs as part of NCR blockade may provide an additional dimension to therapeutic efficacy.

### VISTA: a new horizon in NCR blockade

VISTA, also known as c10orf54, PD-1H [63, 64], DD1α [65], Gi24 [66], Dies1 [67], and SISP1 [68] is a member of the B7 family of NCRs and represents a new target for immunotherapy. Murine VISTA is a type I transmembrane protein with a single IgV domain with sequence homology to its B7 relatives with conserved segments thought to be critical for the IgV stability [69]. However, VISTA also has unique features such as additional cysteine residues in the Ig-V domain, an insertion of a long loop between the C” and D strands, and the absence of a second Ig domain in the ectodomain [69]. These features suggest that VISTA could function as a receptor as well as a ligand. Indeed, structural modeling suggest homology to either PD-1 [63] or PD-L1 [69]. However, VISTA does not cluster with the B7 family at standard confidence limits, suggesting that it is only weakly associated with this family [69]. VISTA is the most conserved among the B7 members. Human VISTA shares 78% identity with murine VISTA according to a Global Alignment Search Tool, an unprecedented sequence identity among NCRs. By comparison, human and murine PD-L1, PD-L2, CTLA-4 and PD-1 share 70%, 63%, 76%, and 59% sequence identity, respectively. In particular, the cytoplasmic tail of VISTA is highly conserved in mice and humans with 86.5% identity and VISTA’s unique features in the extracellular domain are also highly conserved.

The surface expression pattern of VISTA is clearly distinct from CTLA-4, PD-1, and PD-L1. [1] VISTA is expressed on naïve T cells [69] whereas PD-1 and CTLA-4 are not, which may suggest that VISTA functions to restrain T cell activity at an even earlier stage in T cell priming. [2] VISTA is expressed on both T cells and APCs with very high expression on myeloid cells [69]. [3] VISTA is hematopoietically restricted and in multiple cancer models, VISTA was only detected on tumor infiltrating leukocytes and not on tumor cells [70]. This unique surface expression pattern suggests that VISTA may function to restrict T cell immunity at different stages compared to PD-1/PD-L1 and CTLA-4 axes. Importantly, the pattern of VISTA expression is remarkably similar between human and mice (Table 1). Interestingly, VISTA is abundantly stored within intracellular compartments (unpublished), similar to CTLA-4. In fact, the intracellular CTLA-4 compartment is a key component in the stringent regulation of surface CTLA-4. Intracellular CTLA-4 is mobilized to the surface following T cell stimulation in a magnitude which is directly proportional to TCR signaling [5, 71–73]. Comparably, the majority of VISTA is detected within the intracellular compartment of myeloid cells with surface VISTA rapidly endocytosed (unpublished). However, steady, but high, levels of VISTA are maintained on the cellular surface of myeloid cells [69, 70, 74]. Studies investigating the mechanisms underlying intracellular and surface VISTA expression are ongoing.

**Table 1:** VISTA expression levels on human and murine subsets as evaluated by FACS analysis

Cell Type	Surface VISTA expression	
	Human	Mouse
CD4^+^ naïve T cells	+	++
CD4^+^ FoxP3^+^ T_reg_	+	++
CD4^+^ memory T cells	+	++
CD8^+^ T cells	+	+
B cells	-	-
NK cells	-	-
Peritoneal macrophages	N/D	+++
Monocytes	+++	+++
Neutrophils	+++	+++
Dendritic cells	+++	+++

Adapted from Wang et al. and Lines et al

VISTA has been demonstrated to exert both ligand and receptor functions. First, VISTA can function as a ligand to negatively regulate T cell activation. *In vitro*, VISTA on APCs and cell-free VISTA-Ig fusion protein (extracellular domain of VISTA fused with human IgG1 Fc) inhibited CD8^+^ and CD4^+^ T cell at the early stage of activation indicated by suppression of CD69, CD25, CD44, and CD62L [69]. VISTA Ig suppressed the production of IL-2 and IFNγ in both CD4^+^ naïve and memory T cells as well as in CD8^+^ T cells [69]. Unlike the PD-1/PD-L1 pathway, VISTA does not directly regulate T cell response by the induction of apoptosis [69]. VISTA-Ig fusion protein also promotes *in vitro* conversion of naïve CD4^+^ T cells to Tregs in both mouse and human [74, 75]. VISTA expression on Tregs also contributes to the suppression of T cell proliferation in *in vitro* suppression assay [70]. Finally, in mice that were vaccinated with irradiated MCA105 tumor cells to generate immunity, re-challenge with VISTA-overexpressing MCA105 tumor cells lead enhanced tumor growth compared to re-challenge with the VISTA negative parent MCA105 indicating that VISTA expression can overcome protective anti-tumor immunity [69].

Second, VISTA has been demonstrated to function as a receptor on T cells which negatively regulates their activity. VISTA^-/-^ CD4^+^ T cells respond more vigorously than wild type (WT) CD4^+^ T cells to both polyclonal and antigen specific stimulation leading to increased proliferation and production of IFNγ, TNFα, and IL-17A [64]. In addition, VISTA^-/-^ T cells induce exacerbated graft-versus-host disease (GVHD) compared to WT T cells when transferred into F1 recipients [76]. When WT CD4^+^ T cells are stimulated *in vitro* or in vivo in the absence of VISTA on APCs, an anti-VISTA agonist antibody (mam82), which can only target VISTA on the T cell, reduces antigen specific activation [64]. Finally, another anti-VISTA agonist antibody (MH5A) prevents the development of GVHD induced by WT T cells [63] but is ineffective when disease is induced by VISTA ^-/-^ T cells [76]. In addition to T cells, VISTA can also function as a receptor on myeloid cells. Transfection of monocytes from healthy donors to overexpress VISTA led to the spontaneous secretion of inflammatory cytokines IL-8, IL-1β, IL-6, TNFα, and IL-10 [77]. In HIV positive patients, infected monocytes expressed higher amounts of VISTA than healthy monocytes and also spontaneously expressed more TNFα, IL-1β, and IL6 mRNA than healthy monocytes did [77]. VISTA transfected HIV-infected monocytes induced enhanced IFNγ production by antigen-specific autologous T cells compared to vector control and silenced VISTA [77]. In this transfection system with HIV infected monocytes, VISTA-mediated myeloid activation and subsequent T cell activation overshadowed VISTA-driven immunosuppressive functions. VISTA highly conserved cytoplasmic tail does not contain any classic signaling motif. However, it contains potential protein kinase C binding sites as well as proline residues that could function as docking sites for adaptor proteins. In addition, it contains multiple potential serine, threonine, and tyrosine phosphorylation sites. Importantly, the transfection of monocyte with cytoplasmic tail-deficient VISTA abrogated the spontaneous elaboration of cytokine [77], suggesting that signaling through VISTA is both possible and required. The apparent opposing functions of VISTA T cells and monocytes is unresolved and requires further investigation. One possible explanation for this discrepancy is the dysregulated level of VISTA expression in transfected or HIV infected monocytes. Other negative checkpoints inhibitors have been associated with positively or negatively regulating innate cells activity depending on their expression level [78].

In multiple mouse models, VISTA expression is upregulated in the TME and plays a critical role in shaping anti-tumor immunity [70]. Distinct from PD-L1, VISTA expression is restricted to the tumor-infiltrating leukocytes and was not detected on tumor cells. In particular, VISTA expression is specifically upregulated on tumor infiltrating myeloid cells such as myeloid DCs and MDSCs, and on tumor infiltrating Tregs compared to those in the periphery [70]. On MDSCs, VISTA increased almost 10-fold on tumor-infiltrating leukocytes compared to those found in the peripheral lymph node [70]. Importantly, this indicates that tumors with infiltrating immune cells and especially MDSCs may harbor abundant levels of VISTA available for therapeutic targeting.

Anti-VISTA monotherapy reduced tumor growth in multiple pre-clinical models, B16OVA melanoma, B16-BL6 melanoma, MB49 bladder carcinoma, and PTEN/BRAF inducible melanoma [70]. In all models, anti-VISTA enhanced T cell response within the TME as well as systemically leading to increased accumulation, proliferation, CD44 expression, and IFNγ and TNFα production [70]. Additionally, VISTA blockade reduced natural Treg mediated suppression of T cells and diminished tumor-induced differentiation of Tregs [70]. Finally, anti-VISTA reduced tumor-infiltrating MDSCs in the B16OVA and PTEN/BRAF melanoma models [70]. Since abnormal myelopoiesis and accumulation of MDSCs are characteristic of many tumors [79], normalizing the myeloid component offers a new opportunity for immunomodulation by anti-VISTA, a mechanism distinct from other forms of NCR blockade. An anti-human VISTA antibody is currently in phase I clinical trial for evaluation in patients with non-small cell lung cancer among other cancers. The mouse surrogate of anti-human VISTA antibody demonstrated tumor growth inhibition by modulating of the myelomonocytic and T cell compartments in human VISTA knock-in mice [80].

**Fig. 1:**
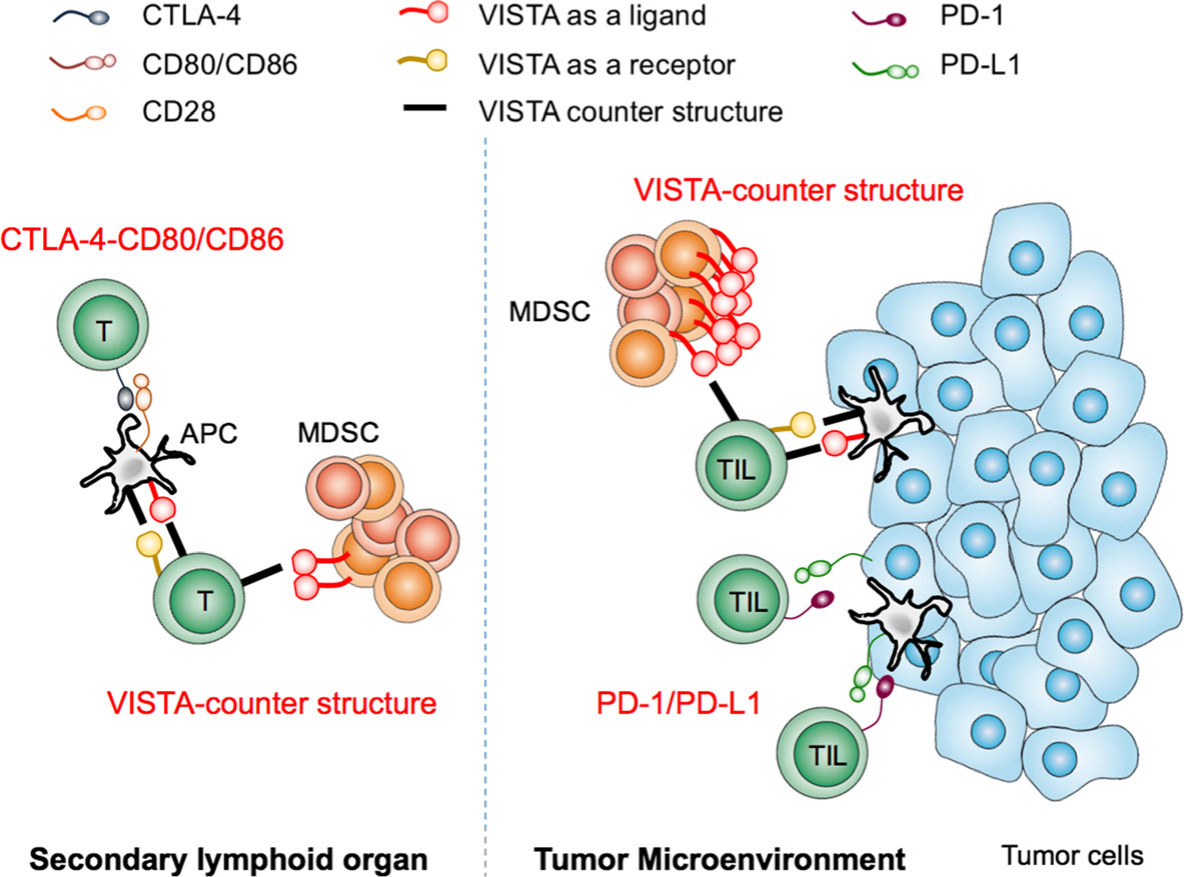
VISTA functions non-redundantly to NCRs currently targeted in clinic. Each NCR occupies distinct temporal and spatial opportunities for blockade to release T cell suppression: [1] VISTA, as a receptor on T cells inhibit early T cell activation while [2] CTLA-4/CD80-86 interaction inhibit post-TCR signaling in secondary lymphoid organs. [3] PD-1/PD-L1 interaction inhibits effector T cells in inflamed tumor tissue. [4] VISTA, as a ligand on MDSCs engages counter structure to inhibit T cells in tumor tissue and secondary lymphoid organs

Important to its clinical potential as a therapeutic target, some aspect of VISTA mediated regulation are unique and therefore, targeting VISTA synergizes with the mechanism of actions of the NCRs currently targeted in the clinic (Fig. 1). Combination antibody blockade (anti-VISTA and anti-PD-1 or anti-PD-L1) synergistically enhanced T cell responses [81]. Ultimately, combination therapy led to reduced tumor growth, enhanced survival, increased IFNγ, tumor necrosis factor (TNFα), and Granzyme B within CD8^+^ T cells further supporting a mechanism of action non-redundant to established NCR blockade in clinic.

## Conclusion

Identification of NCRs as critical mechanisms limiting T cell response and the use of monoclonal antibody blockade to support the development of persistent T cell immunity in the setting of cancer have transformed cancer therapy. Lessons learned from anti-CTLA-4 and anti-PD-1 pathway blockade have encouraged continued discovery and development of NCR blockade while revealing additional opportunities for improvement. As combination immunotherapy continues to rise to the forefront of cancer treatment, targeting VISTA may offer a particularly attractive and unique opportunity for synergism due to its role in restricting very early T cell activation events and high expression on tumor infiltrating MDSCs suggesting that anti-VISTA pathway blockade may occupy a distinct therapeutic compartment.

## Abbreviations

APCs: Antigen presenting cells
CSF-1R: Colony stimulating factor 1 receptor
CTLA-4: Cytotoxic T Lymphocyte Antigen 4
FDA: Food and Drug Administration
GVHD: Graft-versus-host disease
HIV: Human immunodeficiency virus
IDO: Indoleamine 2,3-dioxygenase
ITIM: Immunoreceptor tyrosine-based inhibition motif
ITSM: Immunoreceptor tyrosine-based switch motif
MDSCs: Myeloid-derived suppressor cells
NCR: Negative checkpoint regulator
nTregs: Natural regulatory T cells
PD-1: Programmed cell death 1
PD-L1: Programmed cell death ligand 1
PD-L2: Programmed cell death ligand 2
TCR: T cell receptor
TLR: Toll Like receptor
TME: Tumor microenvironment
VISTA: V-domain Ig Suppressor of T Cell Activation
WT: Wild type
